# Technical guidelines for non-clinical pharmacological research of traditional Chinese medicine (TCM) formulae

**DOI:** 10.1186/s13020-025-01277-w

**Published:** 2025-12-03

**Authors:** Weijie Li, Fangbo Zhang, Ping Wang, Yanqiong Zhang, Peng Cao, Zhijie Ma, Hailan Yao, Jiabo Wang, Hongjun Yang, Haiyu Xu

**Affiliations:** 1 State Key Laboratory for Quality Ensurance and Sustainable Use of Dao-di Herbs, Institute of Chinese Materia Medica, China Academy of Chinese Medical Sciences, Beijing, 100700 China; 2 School of Traditional Chinese Medicine, Capital Medical University, Beijing, 100069 China; 3 Experimental Research Center, China Academy of Chinese Medical Sciences, Beijing, 100700 China; 4 Liaoning University of Traditional Chinese Medicine, Shenyang, 117004 China; 5 State Key Laboratory of Technologies for Chinese Medicine Pharmaceutical Process Control and Intelligent Manufacture, Nanjing University of Chinese Medicine, Nanjing, 210023 China; 6 Beijing Ditan Hospital, Capital Medical University, Beijing, 100015 China; 7 Department of Biochemistry and Immunology, Capital Institute of Pediatrics, Beijing, 100020 China

**Keywords:** Traditional Chinese medicine (TCM) formulae, Non-clinical pharmacology, Integrative pharmacology of TCM, New drug development, Animal model of TCM, Pharmacodynamic evaluation, Molecular regulation

## Abstract

Non-clinical pharmacology of traditional Chinese medicine (TCM) formulae plays a crucial role in elucidating their therapeutic mechanisms. With the integration of cutting-edge disciplines such as systems biology, big data, and artificial intelligence, the research depth and breadth in this field continue to expand. However, technical standards for non-clinical pharmacology research on TCM formulae have not yet been established. To address this, technical guidelines for non-clinical pharmacology research of TCM formulae were formed, aiming to scientifically standardize research content, elevate research quality, and facilitate the mutual translation between clinical and basic research. Guided by integrative pharmacology of TCM and driven by clinical value, these guidelines employ multi-dimensional qualitative pharmacokinetics (PK) and pharmacodynamics (PD) studies alongside quantitative "PK-PD" studies of key components. This approach enables the transition from panoramic description to precise mechanistic research, establishing a complete evidence chain system for the effectiveness of TCM formulae and demonstrating their holistic action characteristics of "multi-component, multi-pathway, multi-target". Meanwhile, the guidelines emphasize that animal models for TCM research should ensure stability and reproducibility. Additionally, pharmacodynamic evaluation is characterized by its measurability and quantifiability. The mechanisms of action at the molecular level should be in-depth and interpretable. These aspects collectively provide guidance for basic non-clinical pharmacology research on TCM formulae and the development of new TCM drugs. The formulation of these guidelines leveraged the research strengths of the drafting units' teams, incorporated literature review findings, and achieved consensus through extensive discussions among multidisciplinary experts. Broad expert opinions from universities, research institutes, and enterprises engaged in new drug development were solicited, ensuring the guidelines' practicality, standardization, scientific rigor, and feasibility.

**Supplementary Information:**

The online version contains supplementary material available at 10.1186/s13020-025-01277-w.

## Introduction

Traditional Chinese medicine (TCM) formulae, serving as the primary clinical treatment approach, are core vehicle for TCM theory. Since the advent of the first animal model of TCM syndrome (*e.g.,* Kidney Yang Deficiency Model) in 1960, non-clinical pharmacology research on TCM formulae has witnessed marked progress in several areas, including animal models of TCM, pharmacodynamic evaluation, pharmacokinetics of TCM formulae, molecular mechanisms research. This advancement has been significantly accelerated in recent years by the deep integration of cutting-edge disciplines, such as systems biology, big data, and artificial intelligence combined with TCM research [1–16]. Building upon this foundation, our team proposed Integrative pharmacology-based TCM (TCMIP) in 2014. The TCMIP is an interdisciplinary science that can establish qualitative and quantitative pharmacokinetics (PK)-pharmacodynamics (PD) correlations through the integration of knowledge from multiple disciplines and techniques and from different PK-PD processes in vivo (direct, indirect, and auxiliary action of TCMs)*.* TCMIP approach has been used to reveal the molecular mechanism from panoramic prediction to precise experimental validation and provides a comprehensive efficacy evidence chain of TCM formulae [17–24]. Exemplified by the Huashi-Baidu Formula (Q-14), modern scientific approaches focusing on antiviral and anti-inflammatory effects have elucidated the anti-Severe Acute Respiratory Syndrome Coronavirus 2 (SARS-CoV-2) virus mechanism of this TCM formula, demonstrating its characteristic "multi-component, multi-pathway, multi-target" holistic action [25].

Under the auspices of the China Association of Chinese Medicine (CACM), the project team collaborated with leading research institutions nationwide to perform the *Technical Guidelines for Non-Clinical Pharmacological Research of Traditional Chinese Medicine (TCM) Formulae*. This research integrated comprehensive literature analysis with multidisciplinary expert consensus through structured deliberations. Underpinned by TCMIP and grounded in traditional TCM knowledge alongside clinical empirical practices, the guidelines employ advanced modern scientific technologies and research methodologies. They facilitate a comprehensive investigation into the interaction mechanisms and intrinsic principles between the complex material system of TCM formulae and intricate biological activities of organisms, achieving through a progressive research strategy from multidimensional PK-PD analysis to quantitation of critical bioactive components. Driven by clinical value orientation, the guidelines mandate that animal models must demonstrate concordance with pathophysiological manifestations and clinical syndrome profiles. Furthermore, the guidelines establish a clinically correlated, multi-tiered and multidimensional pharmacodynamic evaluation matrix. These guidelines will facilitate bidirectional translation between basic research and clinical practice ensuring that pharmacological studies are stable, reproducible in animal models, quantifiable in efficacy evaluation, and interpretable in mechanism investigation via integrative investigation of PK-PD relationships and interactions between macro-scale pharmacodynamics and micro-level mechanisms. The findings may provide technical frameworks for fundamental pharmacological research on TCM formulae and new drug development.

This guidance may contain limitations due to the constraints in the understanding of the drafting team. We sincerely welcome feedback and suggestions from all colleagues. With the ongoing advancement in modern research on TCM compound formulas, this document will be continuously updated, revised, and improved to incorporate relevant research progress promptly.

## General principles

### Constructing a complete evidence chain for the efficacy of TCM formulae

Under the guidance of integrated pharmacology, with “chemical substances-pharmacokinetics-pharmacodynamics” as the core, this guideline advocates comprehensive research integrating macro and micro, global and local perspectives to build a complete evidence chain for the efficacy of TCM formulae. This approach transitions from panoramic characterization to precision mechanism research, not only applying systems biology perspective but also adopting reductionist approaches for mechanism elucidation. The selection of appropriate animal models for pharmacodynamic evaluation is crucial. Based on this, advanced technologies such as systems biology, big data, artificial intelligence, and phenomics should be employed to elucidate the interactions and mechanisms between the material basis of TCM formulae and the organism through direct, indirect, and auxiliary effects. For primary effects, the establishment of quantitative PK-PD models linking multiple components to biological effects is encouraged, to overcome the limitations of classical pharmacokinetic/pharmacodynamic studies, enhance the level of pharmacological research, and promote discipline development.

### Building appropriate animal models and reasonable pharmacodynamic evaluation systems

Non-clinical pharmacological research on TCM formulae should be guided by traditional Chinese medicine theory and based on clinical practice. Animal models should consider both disease and syndrome dimensions to ensure clinical relevance. Improving the operability, feasibility, stability, and reproducibility of animal models is necessary. In pharmacodynamic evaluations, emphasis should be placed on the correlation with clinical efficacy indicators, establishing correlations and hierarchies between research indices to objectify and quantify subjective indicators and enhance the measurability of efficacy. Standardization of animal models and pharmacodynamic evaluations should be pursued to ensure the repeatability of efficacy assessments. On this basis, research into drug metabolism, pharmacokinetics, and molecular mechanisms should be conducted to promote the mutual translation between basic and clinical research in TCM formulae.

### Establishing a scientific, rational, and standardized technical guideline

This document strengthens the classification guidance and proposes corresponding solutions and technical requirements based on different research purposes, scientifically and normatively guiding the non-clinical pharmacological research of non-clinical pharmacology of TCM formulae. The research purposes are divided into basic research and new drug development. Basic research targets TCM formulae with sufficient support from TCM theory and human usage experience (such as classic famous formulas and clinical experience prescriptions). Non-clinical pharmacological research is used to clarify the material basis of pharmacological effects, pharmacological actions, and molecular mechanisms, providing a basis for optimizing formulas and secondary development of TCM preparations. New drug development is divided into the following situations: for TCM compound new drugs with sufficient human usage experience support, non-clinical pharmacological research is not necessary, but it can provide reference for selecting clinical trial efficacy indicators; for TCM compound new drugs derived from experimental research or lacking human usage experience, non-clinical pharmacological research is necessary, divided into two stages: the evaluation of pharmaceutical properties and the registration application. The former focuses on the comparison of key information such as material selection and preparation processes, thus providing a scientific basis for formula optimization and manufacturing process selection; the latter requires normative and scientific effectiveness research data to clarify pharmacological actions and pharmacodynamics characteristics, providing a basis for clinical efficacy research. According to the "*Regulations on the Registration of Chinese Medicine*", new TCM drugs with a clear material basis and a basic understanding of the mechanism of action can be included in the priority review and approval scope. This document provides guidance for related research and serves as a reference for priority review and approval.

## General requirements

### Test samples of TCM formulae

#### Depending on the research objectives, the requirements for TCM formulae test samples (herein after referred to as “test samples”) are as follows:

- a) For basic research, the test samples should generally be consistent with actual clinical use, with clear information on prescription composition, herbal origin, medicinal parts, processing methods, dosages, administration methods, and quality control standards.
- b) When conducting highly specialized identifications such as botanical origin identification, information regarding the identifier or identification institution should be provided to ensure traceability and reliability. Sample preparation may involve laboratory-scale pilot samples or samples produced at pilot-scale or above.

#### New drug research and development can be divided into the following categories:

- a) For TCM compound new drugs supported by sufficient TCM theory and human use experience, the test samples must align with clinical use. Information on herbal origin, medicinal parts, geographical source, harvest period, cultivation or breeding methods, fixed composition and ratio, preparation processes, and manufacturer information must be clearly stated and fixed. Samples for new drug registration reviews should generally be at the pilot scale or larger. If not, sufficient justification must be provided. When administration volume or method is limited, extracts (*e.g.*, concentrated decoctions, effective fractions) may be used for testing.
- b) For TCM compound new drugs derived from experimental studies or lacking human use experience:
- During the developability evaluation stage, the test samples mainly focus on comparative research on prescription composition, herbal origin and geographical source, medicinal parts, processing methods, dosages, and administration methods to provide scientific bases for selecting key information. The comparison must include a demonstration of comparable pharmacological efficacy and safety in relevant non-clinical models. Laboratory-scale samples are acceptable at this stage.
- During the registration application stage, the test sample requirements are consistent with those for TCM formulae supported by TCM theory and human use experience.

In both basic research and studies aiming for priority review, the material basis and mechanism of action of the TCM formulae should be clarified. Therefore, systematic chemical component analysis of the test samples should be conducted to ensure a clear understanding of the material basis. Detailed sample requirements are provided in Supplementary Table S1.

### Administration route, dose, and frequency

The requirements for administration routes, doses, and frequency are detailed in Supplementary Table S2.

### Animal experimental design, sample size, and control studies

#### Experimental design

The design should follow principles of randomization, control, repetition, scientific rigor, and adhere to the 3Rs principle: Reduction, Replacement, and Refinement. Commonly used experimental animals include Caenorhabditis elegans (nematodes), zebrafish, mice, rats, guinea pigs, golden hamsters, rabbits, and beagle dogs.

#### Sample size

The number of experimental animals may vary depending on the species and research purpose. Especially during the developability evaluation stage, the number of animals may be appropriately reduced. In principle, animals should be evenly distributed between males and females. If the intended clinical application is gender-specific, the corresponding gender of animals may be selected.

#### Control studies

The experimental design should consider the use of reasonable blank controls, negative controls, and positive controls. The selection of the positive control drug is crucial and should follow the principle of "comparable within the same category, universally recognized as effective" or "clear efficacy, comparable in effects." In other words, the chosen positive control drug should belong to the same category as the test drug, meaning it has similar pharmacological actions or therapeutic mechanisms. During the design and implementation of the experiment, it should be ensured that the test drug and the positive control drug are comparable in terms of dosage, administration route, treatment cycle, and other factors. The positive control drug should have clear efficacy and be widely recognized as effective for specific diseases, syndromes, or symptoms. Depending on the experimental purpose and animal model, an appropriate positive drug should be chosen. For example, in experiments conducted on disease animal models, it is recommended to choose a first-line western medicine for disease treatment as the control; in experiments conducted on syndrome animal models, a widely recognized effective TCM compound should be chosen as the control; in experiments conducted on disease-syndrome combined animal models, it is recommended to choose a first-line western medicine for disease treatment or a widely recognized effective TCM compound as the control; when a TCM compound is used as an adjunctive treatment, which is added to Western medicine to improve clinical symptoms and enhance quality of life, an effective or widely used adjunctive drug can be chosen as the control.

## Main research content

### Selection and evaluation of non-clinical pharmacodynamic models

#### Construction and evaluation of animal models

##### Requirements for experimental animals

To ensure the reliability, accuracy, and reproducibility of experimental results, appropriate animal species should be selected based on the research objectives, taking into account multiple factors such as species consistency, genetic background, microbiological status, age, body weight, gender, health status, ethical and legal requirements, and rearing conditions. Experimental animals must comply with national regulatory standards and provide the following information: producer name, production license number, breed and strain, quality grade, specifications, quality inspection date, and inspection institution.

Ethical approval from the institution's ethics committee must be obtained prior to conducting animal experiments, with the relevant approval number recorded. Animals should undergo adaptive housing to acclimate to environmental and lifestyle conditions before model establishment. The use and research involving experimental animals should comply with the 3Rs principle: Reduction, Replacement, and Refinement.

##### Selection and evaluation of animal models

Animal models can be classified into disease animal models, syndrome animal models, and disease-syndrome combined animal models. Based on the research purpose, clinical positioning, and prescription characteristics of the TCM compound, animal models should be scientifically and rationally selected and used.

To explore the pharmacological actions and molecular mechanisms of TCM formulae against diseases, widely accepted and extensively used disease animal models within the field should be employed. In selecting disease models, attention should be given to their anatomical, pathological, physiological, and metabolic similarities to human clinical diseases. Additionally, the reproducibility, reliability, and economic feasibility of disease models should be evaluated (Refer to Supplementary Table S3). The model should ideally mimic key clinical features of the syndrome to increase the predictive value of the efficacy findings. If possible, it is crucial to assess the animal model by selecting biomarkers for clinical relevance as key clinical features, such as specific inflammatory cytokines, metabolic profiles, and imaging markers. When selecting an animal model, in addition to pathophysiological relevance to the human disease, careful consideration must be given to the stage of disease progression that the model represents. This is particularly crucial for TCM formulae intended for a specific phase of complex diseases. The model should mimic the clinical disease stage for which the formula is clinically.

For syndrome animal models, construction methods include TCM etiology modeling, drug-induced modeling, direct pathological modeling, and combined etiology-pathology models. To investigate the efficacy and molecular mechanisms of TCM formulae in treating syndromes, relatively mature and well-established syndrome models should be used. Syndrome models should correspond well with TCM clinical manifestations, including main symptoms, secondary symptoms, tongue and pulse diagnosis, and also reflect pathological, physiological, functional, and metabolic similarities to clinical syndromes. If scientific evaluation of the syndrome model has already been conducted based on primary symptoms (core evidence), causes (positive evidence), treatment effects (counter-evidence), related factors (supportive evidence), and objective indicators (supportive evidence), the rationale for model selection becomes even stronger and should be prioritized. Additionally, reproducibility, reliability, and cost-effectiveness of syndrome animal models must also be evaluated. (Please see Supplementary Table S3).

The combination of disease and syndrome refers to the integration of Western medicine's differentiation of diseases with traditional Chinese medicine's differentiation of syndromes, and it is one of the main models in current TCM clinical diagnosis and treatment. To explore the effects and molecular mechanisms of TCM formulae on the treatment of disease-syndrome combinations, a disease-syndrome combined animal model should be selected for research. There are mainly three approaches to developing disease-syndrome combined animal models: first, the integration of TCM and Western medicine by using multi-factor composite modeling, which involves establishing a disease animal model based on modern medical etiological replication and simultaneously creating a syndrome model based on TCM etiology and pathogenesis (such as the six pernicious influences, diet, overwork, emotions, etc.), so that the model animal exhibits both disease and syndrome (symptom) characteristics; second, exploring TCM syndromes based on Western medicine disease models, which involves dynamically observing and collecting the symptoms, signs, and laboratory indicators of experimental animals during or after the creation of the Western disease model, analyzing and summarizing the dynamic evolution and characteristics of the "syndrome" during the disease formation process, thus identifying a specific disease-syndrome combined model; third, exploring Western medicine disease types based on syndrome models, which involves using traditional TCM etiological modeling methods to simulate environmental factors for the animals, observing macroscopic characteristics, testing physicochemical indicators, and using representative prescriptions for verification, and after establishing the syndrome animal model, searching for relevant clinical diseases for verification. When selecting a disease-syndrome combined animal model, it is necessary to ensure that the main symptoms, secondary symptoms, tongue, and pulse conditions correspond to TCM syndromes, and that it also aligns with Western medicine’s disease characteristics in terms of pathology, physiology, function, metabolism, and structure. If the syndrome animal model has already undergone scientific evaluation based on symptom-related evidence (core evidence), etiology-related evidence (positive evidence), treatment-related evidence (counter-evidence), associated factor evidence (supportive evidence), and objective indicator evidence (supportive evidence), then the rationale for selecting this model is well-founded and it should be given priority. Meanwhile, the reproducibility, reliability, and cost-effectiveness of the syndrome model should also be evaluated. (Please see Supplementary Table S3).

Animal models should be further validated in clinical research. If discrepancies between the animal model and human disease characteristics arise during model construction, it is necessary to analyze the nature and extent of these differences and to assess the scientific value of the model appropriately.

#### Construction and evaluation of in vitro pharmacological models for TCM formulae

At present, in vitro pharmacological evaluation models for TCM formulae include isolated organs, isolated tissues, organoids, cells, organelles, enzyme activity assays, and molecular interaction models. To enhance scientific rationality, in vitro models should be selected based on key pharmacological pathways, mechanisms of action, and molecular targets identified from clinical applications or animal models, ensuring consistency or relevance between in vitro and in vivo studies. Different levels of in vitro models require attention to different evaluation factors. Detailed recommendations are provided in Supplementary Table S4.

The administration methods in vitro pharmacological studies can be divided into mixture administration and single-component administration. Mixture administration can further be categorized into direct drug addition, drug-containing serum method, drug-containing intestinal absorption liquid method, and so on [26–31]. The advantages and disadvantages of each administration method are detailed in Supplementary Table S5. From the perspective of administration methods, the key is consistency with the pharmacological material basis of target tissues/cells in clinical or animal models. Advanced technologies such as organoids and microfluidic chips can be used to develop in vitro "pharmacokinetics-pharmacodynamics" dynamic composite new models that align with the complex system characteristics of TCM. To ensure the accuracy and repeatability of the experiment, the in vitro pharmacological experiment should involve repetition of sample sizes and trials. Experimental groups should include a blank control group, a negative control group, and a solvent control group, with a positive control drug group set up when necessary. The drug concentration should comprehensively consider clinical human equivalent doses, preliminary test results, and other experimental information. Generally, the test drug should be designed with at least three different concentrations to explore the dose–response relationship. The selection of observation and detection indicators should be based on in vivo animal experiments, with sensitivity and specificity as the basic principles, while complementing and enriching the results of in vivo studies to analyze the potential mechanisms of action of TCM deeply.

Monomer component administration involves evaluating the pharmacological effects and molecular mechanisms of individual components from TCM formulae using in vitro pharmacological models. Based on the identification of key action pathways, pharmacodynamic processes, pharmacological effects, or active substances of the TCM formula through animal models, in vitro pharmacological studies of individual components are then conducted.

### Pharmacodynamic evaluation of TCM formulae

Traditional Chinese medicine compound prescriptions exhibit phenomena such as "one formula with multiple effects" and "one formula with multiple functions". The pharmacodynamic evaluation indicators for TCM formulae include phenotypic indicators, physiological parameter indicators, biochemical indicators, pathological indicators, imaging indicators, omics evaluation indicators, and TCM syndrome symptom-related indicators, as detailed in Supplementary Table S6.

For different dimensions and levels of indicators, research should be conducted on the correlations between these indicators in order to identify objective substitutes for unobservable and unmeasurable indicators such as subjective symptoms, tongue and pulse, thus expanding the measurability of TCM pharmacodynamic evaluation indicators. At the same time, the research on the correlations between indicators from different dimensions and levels should be strengthened, establishing a reasonable hierarchical structure for the pharmacodynamic indicator system and conducting layer-by-layer analysis. Fuzzy mathematics and statistical methods should be introduced to analyze the weights and correlations of complex, interrelated indicators, so that the pharmacodynamic indicators are not simply a mechanical combination or simple sum, but rather an inseparable whole where all elements are interrelated.

For key pharmacodynamic indicators, the pharmacodynamic evaluation indicators of TCM formulae should undergo a comprehensive evaluation in terms of specificity, sensitivity, accuracy, repeatability, and cost-effectiveness, and the significance, applicable scope, levels, and thresholds of the selected indicators should be explained to achieve broad recognition and adoption. Additionally, the "dose–effect" and "time-effect" relationships of TCM formulae should be explored to provide scientific evidence for determining the drug dosage, frequency, and treatment cycle. At the same time, for cases where the "dose–effect" relationship of certain pharmacodynamic indicators is not significant or irregular, a scientific understanding and reasonable analysis should be conducted.

### Metabolism and pharmacokinetics of TCM formulae

#### Overview

The study of metabolism and pharmacokinetics of TCM formulae represents an important advancement in understanding the spatiotemporal distribution of chemical substances within the body. It serves as a vital bridge linking the chemical composition of TCM formulae with their pharmacodynamics and mechanisms of action. This document aims to guide such research to better support non-clinical pharmacological studies and to elucidate the molecular mechanisms of TCM formulae efficacy. In terms of experimental design, efforts should be made to maintain consistency with the relevant content outlined in "Pharmacodynamic Evaluation of TCM formulae" section, including the test samples of TCM formulae, routes of administration, dosage, and frequency of administration. The use of the same animal models employed in pharmacodynamic studies is encouraged for conducting metabolic and pharmacokinetic research on TCM formulae. However, when applying specific disease models proves challenging, healthy adult animals-such as mice, rats, rabbits, guinea pigs, dogs, miniature pigs, and monkeys-may also be used for such studies.

#### Research content

The primary content of metabolism and pharmacokinetics studies of TCM formulae includes systematic analysis of in vivo migrating components, multi-component PK studies, and the exploration of PK-PD relationships between multiple components and biological effects. Systematic analysis of in vivo migrating components focuses on investigating the metabolic reactions and patterns of TCM formulae within the body, including "qualitative (structural) and quantitative (concentration) metabolic transformations", and is primarily qualitative and semi-quantitative in nature. Sampling time points should be carefully designed based on the pharmacodynamic characteristics of the TCM formula, particularly its "time-effect" relationships. Collected samples typically include gastrointestinal contents, serum, feces, urine, and various organs (such as the heart, liver, spleen, lungs, kidneys, etc.). High-resolution and high-sensitivity chromatographic-mass spectrometric techniques, such as ultra-performance liquid chromatography-quadrupole time-of-flight mass spectrometry (UPLC-Q/TOF–MS) or ultra-high performance liquid chromatography-quadrupole-electrostatic field orbitrap high resolution mass spectrometry (UPLC-LTQ-Orbitrap-MS), are generally used to systematically identify and analyze the migrating components in vivo, including both prototype compounds and their metabolites for qualitative and quantitative analysis. When conducting systematic analysis of in vivo migrating components, attention should be paid to methodological validation, including control experiments (such as blank solvents, negative in vivo biological samples, and drug-containing in vivo biological samples), reproducibility of retention time deviations, relative abundance in standard spectra, and signal-to-noise ratios of detected ions.

#### Analytical methods

Common analytical methods used in the pharmacokinetic studies of multi-component TCM formulae mainly include chromatographic techniques, such as high-performance liquid chromatography (HPLC), gas chromatography (GC), and chromatography-mass spectrometry (*e.g.*, LC–MS, LC–MS/MS, GC–MS, GC–MS/MS). For the quantitative analysis in TCM formula pharmacokinetic studies, methodologies should refer to the *Technical Guidelines for Non-clinical Pharmacokinetic Studies of Drugs (2024)*, covering aspects such as accuracy, precision, specificity, sensitivity, reproducibility, stability, standard curves, extraction recovery, quantification range, biological matrices, and matrix effects. Commonly used pharmacokinetic parameter calculation software includes 3P87/3P97 Practical Pharmacokinetic Calculation Programs, DAS statistical software, WinNonlin, and Kinetica, which can be applied to calculate statistical moment parameters from drug concentration–time curves to describe the pharmacokinetic characteristics of multi-component TCM formulae. In addition, absorption, distribution, metabolism, and excretion (ADME)/PK prediction models are crucial for TCM pharmacokinetic research. Developing ADME/PK prediction methods tailored to the characteristics of TCM and introducing appropriate pharmacokinetic models [such as Physiologically Based Pharmacokinetic‌ (PBPK) and Population Pharmacokinetics (PopPK) models) are important steps in building a human PK prediction system, thereby better supporting the clinical application of TCM formulae.

#### Correlation studies

The PK/PD relationship of TCM formulae are often characterized by complexity and non-linearity. This includes multidimensional qualitative PK-PD association studies and quantitative PK-PD network models involving multiple components of TCM formulae. In panoramic, multidimensional qualitative PK-PD research, the reverse pharmacokinetics approach can be applied to infer the pathways and mechanisms of action of TCM formulae based on their pharmacokinetic characteristics. If the in vivo migrating components (both prototypes and metabolites) of a TCM formulae are primarily distributed in plasma and target tissues, it can be inferred that the compound exerts its effects through direct action. In such cases, a multidimensional qualitative network can be constructed linking "in vivo migrating components-target sites-regulatory pathways-syndrome-related effects". Conversely, if these components are mainly distributed in non-target tissues, it is likely that the compound exerts its effects through indirect action, possibly involving cross-organ (or cross-tissue) regulation. In such cases, identifying intermediate mediators becomes critical. Clarifying the regulatory roles and relationships between TCM components and these intermediates enables the construction of a multidimensional qualitative network model linking “in vivo migrating components-intermediate mediators-cross-organ regulatory effects-regulatory pathways-syndrome-related effects." In terms of auxiliary regulatory effects, research can focus on drug interactions between TCM formulae and chemical drugs, as well as interactions within the TCM compound itself (*e.g.*, between herbs, between active parts, or between components). Studies can also concentrate on interactions involving pharmacokinetic markers, exploring underlying mechanisms such as transporters and drug-metabolizing enzymes.

In quantitative PK-PD modeling studies of multi-component network effects in TCM, based on a complete evidence chain system of “chemical substances-targets-network targets-syndrome-related effects,” the pharmacokinetic markers of a TCM formula can be systematically identified by integrating factors such as chemical composition content, in vivo exposure levels, bioactivity strength, and topological features within biological networks. Additionally, the major biomarkers related to syndrome effects can be determined. Subsequently, advanced computational techniques that align with the characteristics of TCM data-such as entropy-based neural network multi-objective optimization and Least Angle Regression (LARS) multi-objective optimization based on an improved ant colony algorithm-can be employed to innovatively construct quantitative PK-PD models of multi-component network effects, can be applied to innovatively establish quantitative PK-PD models for the network effects of multi-component TCM formulae. These models can then be linked with the total statistical moment algorithm to quantitatively describe the “dose-time-effect" relationship of TCM formulae, thereby providing an in-depth explanation of how TCM formulae exert their integrated pharmacodynamic effects over time and dose.

### Molecular mechanism research of TCM formulas

Big data could be used to analyze complex, high-dimensional datasets from pharmacological studies and clinical records to identify novel patterns and associations. The study of the molecular mechanisms underlying the actions of TCM formulae is guided by the strategy of integrative pharmacology. It utilizes emerging frontier technologies such as systems biology, big data and artificial intelligence, single-cell omics, and spatial omics to explore the complex molecular mechanisms of TCM formulae from a holistic perspective. At the same time, it focuses on the primary therapeutic effects of the formulas, delving deeply into the regulatory roles and relationships between the complex multi-component systems of TCM formulae and their principal therapeutic effects through both direct and indirect mechanisms. This approach enables a transition from a panoramic understanding of the complex molecular mechanisms to a precise and in-depth mechanistic study, forming an integrated research model that combines systems theory, reductionism, and cybernetics. The research on the molecular mechanisms of TCM formulae includes panoramic molecular mechanism prediction, the identification of the primary therapeutic effects and excavation of related molecular mechanisms, multi-dimensional "PK-PD" qualitative association studies based on three regulatory pathways, and quantitative modeling of multi-component network effects in the "PK-PD" framework.

The panoramic prediction of molecular mechanisms underlying TCM formulae can be achieved through several approaches. These include utilizing omics technologies such as transcriptomics, proteomics, metabolomics, as well as emerging methods like single-cell omics and spatial omics, to perform high-throughput sequencing or detection on animal models (e.g., negative control group, model group, and treatment group), followed by bioinformatics analysis to identify differential molecules associated with the efficacy of TCM formulae. Alternatively, molecular bases related to the efficacy of TCM formulae can be obtained from existing sequencing results of clinical or animal samples, although attention should be given to the relevance and scientific rationality compared to animal studies already conducted. Another approach is to retrieve molecular information from disease phenotype databases such as MalaCards Disease Database (MalaCards), Online Mendelian Inheritance in Man ‌(OMIM), and Human Phenotype Ontology ‌(HPO), as well as traditional Chinese medicine databases like SymMap, while emphasizing the completeness and standardization of collected data, and reasonably evaluating the sources and screening processes to ensure scientific reliability.

Based on panoramic mechanism prediction, the principal therapeutic effects of TCM formulae and their associated molecular mechanisms can be identified by employing biological network analysis techniques, such as evaluating features of nodes and edges, network cohesion, divisibility, modularity, and motifs, combined with pharmacodynamic characteristics. To validate the key molecular mechanisms underlying the disease-modifying effects of TCM formulae, molecular biology methods such as polymerase chain reaction (PCR), Western blotting, and enzyme-linked immunosorbent assay (ELISA) should be utilized to ensure the reliability of research results.

Based on the “PK-PD” correlation model between multiple components of TCM and their biological effects, the action pathways of TCM formulas can be classified into direct regulation, indirect regulation, and auxiliary regulation, each with distinct research approaches and content (Please see Supplementary Table S7 for recommendations). Direct regulation involves identifying and analyzing target-reaching components, evaluating their bioactivity, determining direct targets, studying regulatory relationships, and elucidating the synergistic mechanisms among multiple components. Indirect regulation focuses on identifying cross-organ regulation phenomena, discovering intermediary substances, studying their cross-organ mechanisms, and exploring the interactions between formula components and intermediary substances. Auxiliary regulation involves confirming such effects, conducting PK-PD synergy studies, and investigating the molecular mechanisms underlying the auxiliary roles. These three regulatory pathways together form an evidence chain linking "chemical substances-action targets-network targets-syndrome-related effects," which supports the improvement of non-clinical pharmacological research and expands the scientific perspective of TCM formula studies.

While this work provides a framework for the non-clinical pharmacological evaluation of TCM formulae, it is recognized that the drug metabolism and pharmacokinetics (DMPK) research of high-molecular-weight active constituents, such as polysaccharides, peptides, and proteins, requires specialized approaches. These macromolecules often exhibit unique behaviors regarding intestinal absorption, distribution confined largely to the vascular compartment, and complex metabolic fates. Future research updates may aim to develop and standardize methods for tracing these components, assessing their bioavailability, and understanding in vivo processes, which will be critical for fully elucidating the mechanism of action of many TCM formulae. Based on a panoramic mechanism analysis, techniques such as sterile mouse microbiota colonization and in vitro "PK-PD" dynamic composite models can be used to clarify the primary action pathways and major disease-syndrome effects of TCM formulae. On this basis, further identification of pharmacokinetic biomarkers, core targets, etc., of TCM formulae can be carried out through techniques such as component knock-out/knock-in, gene knock-out/overexpression, and by analyzing chemical component content, in vivo exposure, bioactivity intensity, and biological network topological characteristics. At the same time, endogenous biomarkers (groups) related to efficacy can be selected, which should have high sensitivity, good accuracy, and strong specificity, capable of reflecting the differences in pharmacodynamic activity of different effective component combinations. This will help establish a quantitative "PK-PD" correlation model for the multi-component network effects of TCM and provide an in-depth explanation of the "dose-time-effect" relationship between TCM formulae and overall biological effects. It should also be recognized that the quantitative "PK-PD" correlation model for multi-component network effects of TCM is different from the "PK-PD" correlation of single components, as it has nonlinear, multi-target, and discrete characteristics.

## Application of this document

### Guidance for basic research

In general, the clinical practice of TCM serves as the source and foundation for the non-clinical pharmacological research of TCM formulae. During the process of clinical practice, it is important to fix and clarify relevant information about the TCM formulation. In basic research, the basic information of the TCM formulation sample should remain consistent with its clinical application, ensuring that key details such as the prescription composition, medicinal herb origin, medicinal parts, processing specifications, and preparation methods are clearly defined. This also guarantees the uniformity and stability of the quality of the TCM formulation sample and systematically characterizes its material foundation. Based on the information about the dosage, frequency, and duration of administration used in clinical practice, reasonable dosages, administration frequencies, and cycles for animal models should be designed accordingly. Furthermore, based on the clinical understanding of how the TCM formulation treats or improves syndromes and diseases, including their pathological, physiological, and biological foundations, this information should guide the selection of experimental animals and animal models. Specifically, a systematic pharmacodynamic evaluation of the TCM formulation should be conducted, including both macro-level pharmacodynamic indicators and micro-level molecular indicators, subjective and objective pharmacodynamic indicators, primary and secondary pharmacodynamic indicators, and short-term and long-term pharmacodynamic indicators. This comprehensive evaluation will assess the pharmacodynamic characteristics of the TCM formulation and consider its relevance to clinical efficacy.

On this basis, studies can be conducted to explore the complex mechanisms of TCM formulas, including their drug metabolism and pharmacokinetics (DMPK) and molecular mechanisms of action. The DMPK of TCM formulas refers to the spatiotemporal distribution of their chemical constituents in vivo and enables qualitative and quantitative exploration of the metabolic disposition and patterns of complex substances within the body. DMPK also serves as a critical bridge linking the chemical characteristics of TCM formulas to their pharmacological effects and mechanisms. Based on the DMPK characteristics of TCM formulas, their pathways and mechanisms of action can be deduced in reverse. For migrating components of TCM formulas that show high exposure levels in target organs/tissues, direct regulatory effects should be primarily considered. For those with high exposure in non-target organs/tissues, indirect regulatory mechanisms should be emphasized. For clearly identified active constituents, it is important to assess whether other components interact with them (*i.e.*, drug-drug interactions), with a focus on investigating auxiliary regulatory effects.

In the study of the molecular mechanisms of TCM formulation effects, the system analysis of complex mechanisms can be approached through pathways such as direct regulation, indirect regulation, and auxiliary regulation. Different pathways imply different research ideas and contents, as follows: In the aspect of direct regulation, evidence acquisition and relevance evaluation can be carried out through the exploration of disease-symptom effect-related molecular mechanisms, identification of target components, discovery of action targets, and research on regulatory relationships. In the aspect of indirect regulation, evidence acquisition and relevance evaluation can be carried out through the determination of inter-organ regulatory effects, discovery of intermediary mediators, research on inter-organ regulatory effects and mechanisms of intermediary mediators, and the research on the regulatory relationship between TCM formulation components and intermediary mediators. In the aspect of auxiliary regulation, evidence acquisition and relevance evaluation can be carried out through the confirmation of auxiliary regulatory effects, the establishment of multi-component synergistic “PK-PD” models, and the study of molecular mechanisms of regulatory relationships. Through the systematic analysis of these three pathways, a comprehensive multi-dimensional evidence chain system is formed, linking the "chemical fingerprint—metabolic fingerprint—action target—regulatory biological network—disease-symptom effect" of TCM formulations. On this basis, key pharmacological substances, main action pathways, core molecular mechanisms, and major disease effects of TCM formulations can be identified, and a quantitative model of TCM multi-component network effects “PK-PD” can be constructed, achieving a transition from panoramic to precise, and from qualitative to quantitative research on the molecular mechanisms of TCM formulations. At the same time, attention should be paid to evaluating the credibility of the effectiveness of TCM formulations by assessing the sources, objectivity, reliability, and completeness of the evidence, thus enhancing the level of non-clinical pharmacological research on TCM formulations.

### Guidance for new TCM drug development

According to the *"Traditional Chinese Medicine Registration Classification and Application Documentation Requirements"*, new traditional Chinese medicines are categorized into innovative traditional Chinese medicines, improved traditional Chinese medicines, classic ancient prescriptions, and medicines with the same name and prescription. The "three-in-one" evidence system (integration of TCM theory, human use experience, and clinical trials) is the core guiding principle for the research and development of new TCM prescription. Non-clinical pharmacological research on TCM formulations should be integrated with the "three-in-one" evidence system, fully supporting the development of new TCM formulations under the guidance of "preclinical thinking". For TCM formulations that are well-supported by TCM theory and human use experience, pharmacodynamics studies can be exempted when applying for clinical trials and/or market authorization. However, non-clinical pharmacological research on TCM formulations should be used to provide reference for selecting clinical trial efficacy indicators, promoting translational research between basic and clinical studies. For TCM formulations derived from scientific research or those lacking sufficient human use experience, such as research formulas, effective parts, component-based medicines, or new combinations of ingredients, non-clinical pharmacological studies should be conducted. The non-clinical efficacy evidence of these formulations serves as important support for early hypothesis testing and can provide important efficacy information for the initiation of clinical trials.

According to the "*Special Provisions on the Registration and Administration of Traditional Chinese Medicine*", new traditional Chinese medicines with a clear understanding of their medicinal substance base and basic mechanisms of action are included in the list of those subject to priority review and approval. Through non-clinical pharmacological research on TCM formulations, particularly in-depth molecular mechanism studies, the pharmacological substances of the formulations can be systematically identified, providing a basis for the rational selection of quality control components and the scientific establishment of quality standards. This helps to clarify the action targets and regulatory molecular networks of the TCM formulations, providing scientific evidence for their precise clinical application. Therefore, non-clinical pharmacological research on TCM formulations not only enhances the development level of new TCM formulations but also provides valuable reference for priority review and approval.

The detailed research contents related to non-clinical pharmacological studies on TCM formulations for basic research and new drug development are outlined in Table 1.

**Table 1:** Application of non-clinical pharmacological studies of TCM formulae in basic research and new drug development

Main content	Related requirements			Basic research	New drug development of TCM formulae			
					Formulas supported by TCM theory and human use experience	Formulas derived from experimental research or lacking human experience		Priority approval circumstances
						Developability evaluation stage	Registration and approval stage	
Sample information of TCM formulae	Basic information	Define prescription composition, herbal origin, processing specifications		+	+	+	+	+
	Preparation process/scale	Define extraction, drying, preparation process information; Pilot scale or above		+	+	+	−	+
		Define extraction, drying, preparation process information; Pilot scale or above for registration		+	+	+	+	+
	Material basis	Clarify material basis through qualitative and quantitative analysis		+	−	−	−	+
Dosing regimen	It should be consistent and relevant to clinical medication in terms of administration route, dosage, frequency, and other factors			+	+	−	+	+
	It should provide scientific evidence for the clinical medication to be carried out in terms of administration route, dosage, frequency, and other factors			+	+	+	+	+
Basis for animal model selection	Similarity evaluation			+	+	+	+	+
	Reproducibility evaluation			+	+	+	+	+
	Reliability evaluation			+	+	+	+	+
	Economic feasibility evaluation			+	+	+	+	+
Construction and evaluation of pharmacodynamic indicator system	Primary pharmacodynamic indicators	General phenotypic indicators		+	+	+	+	+
		Physiological and Biochemical Indicators		+	+	+	+	+
		Pathological Morphology Indicators		+	+	+	+	+
		TCM Syndrome-Related Indicators		+	+	+	+	+
		Disease-Syndrome-Related Molecular Indicators		+	+	+	+	+
		Omics Technologies and Biomarker Indicators		+	+	+	+	+
Molecular mechanism research of TCM formulae	Analysis of regulatory pathways	Direct modulation	Exploration of Molecular Mechanisms Related to Therapeutic Effects of Syndromes and Diseases	+	−	−	−	+
			Identification of Target-Reaching Components	+	−	−	−	+
			Discovery of Direct Targets	+	−	−	−	+
			Multidimensional Regulatory Relationship Study	+	−	−	−	+
		Indirect modulation	Identification of Cross-Organ Regulatory Effects	+	−	−	−	+
			Discovery of Intermediate Mediators	+	−	−	−	+
			Research on Mediator Cross-Organ Regulatory Mechanisms	+	−	−	−	+
			Study of Regulatory Relationships between TCM Components and Mediators	+	−	−	−	+
		Auxiliary modulation	Confirmation of Auxiliary Modulation Effects	+	−	−	−	+
			Multi-Component PK-PD Synergistic Effect Study	+	−	−	−	+
			Multidimensional Molecular Regulatory Studies	+	−	−	−	+
	Construct a Multidimensional Qualitative Correlation Network of "Chemical Fingerprint-Metabolic Fingerprint-Action Targets-Regulatory Biological Networks-Therapeutic Effects" Based on Three Mechanistic Pathways, Forming a Complete Evidence Chain for the Efficacy of Traditional Chinese Medicine Formulas			+	−	−	−	+
	Establish Quantitative PK-PD Correlation Study of Multi-Component Network Targets Based on Key Active Components and Major Therapeutic Effects			+	−	−	−	+

+ indicates recommended research

−indicates research that may not be necessary

## Supplementary Information

Supplementary material 1

## Data Availability

No datasets were generated or analysed during the current study.

## Abbreviations

ADME: Absorption, distribution, metabolism, and excretion
CACM: China Association of Chinese Medicine
DMPK: Drug metabolism and pharmacokinetics
ECG: Electrocardiogram
ELISA: Enzyme-linked immunosorbent assay
GC: Gas chromatography
H&E: Hematoxylin–eosin
HPLC: High-performance liquid chromatography
Q-14: Huashi Baidu formula
HPO: Human phenotype ontology
TCMIP: Integrative pharmacology-based TCM
LARS: Least angle regression
MalaCards: MalaCards disease database
OMIM: Online Mendelian Inheritance in Man
PD: Pharmacodynamics
PK: Pharmacokinetics
PBPK: Physiologically Based Pharmacokinetic
PCR: Polymerase chain reaction
PopPK: Population pharmacokinetics
SARS-CoV-2: Severe acute respiratory syndrome coronavirus 2
TCM: Traditional Chinese medicine
UPLC-LTQ-Orbitrap-MS: Ultra-high performance liquid chromatography-quadrupole-electrostatic field orbitrap high resolution mass spectrometry
UPLC-Q/TOF–MS: Ultra-performance liquid chromatography-quadrupole time-of-flight mass spectrometry
